# Determining the efficacy of visual inspections at detecting non-biosecurity–compliant goods

**DOI:** 10.1167/jov.24.8.8

**Published:** 2024-08-16

**Authors:** Kambiz Esfandi, Saeedeh Afsar, Kate Richards, Duncan Hedderley, Samuel D. J. Brown, Adriana Najar-Rodriguez, Mike Ormsby

**Affiliations:** 1 The New Zealand Institute for Plant and Food Research Limited, Auckland, New Zealand; 2 The New Zealand Institute for Plant and Food Research Limited, Palmerston North, New Zealand; 3 The New Zealand Institute for Plant and Food Research Limited, Christchurch, New Zealand; 4 Ministry for Primary Industries - Manatū Ahu Matua, Wellington, New Zealand

**Keywords:** phytosanitary import inspection, quarantine pests and diseases, visual search, signal detection theory

## Abstract

Examination of imported commodities by trained inspectors searching for pest organisms is a common practice that phytosanitary regulatory agencies use to mitigate biosecurity risks along trade pathways. To investigate the effects of target size and color on the efficacy of these visual assessments, we affixed square decals to polystyrene models of mandarins. Sample units of 100 model fruit containing up to 10 marked models were examined by inspectors. Six sizes in six shades of brown were tested across two prevalence levels. The experiment consisted of five inspection rounds where 11 inspectors examined 77 sample units within an allocated time. The probability that decals were detected increased with mark size and color contrast. Smaller, low-contrast marks were mainly missed. The prevalence rate did not affect the detectability. Through the experiment, the false-positive rate dropped from 6% to 3%, whereas false-negative rates were constant throughout. Large, dark targets were readily found with a mean recall of >90%, whereas small, pale marks had a mean recall of 9%. Increased experience made inspectors more competent at recognizing decals, reducing the false positive rate. However, constant false-negative rates indicate that experience did not prevent inspectors from overlooking targets they could not perceive.

## Introduction

Inspecting commodities for pests is a procedure that biosecurity agencies use to prevent the movement of unwanted organisms along trade pathways (Eschen et al., 2015; IPPC, 2019). These procedures involve manual and visual inspection of a sample of imported goods and products by trained inspectors. Visual inspection is a deliberate, exacting process that requires a significant mental processing, concentration, information transmission, and extensive use of short-term and long-term memory (Dillard et al., 2019; See, 2012; Warm, Parasuraman, & Matthews, 2008). The inspection process is made more difficult by the rare occurrence of pests (Venette, Moon, & Hutchison, 2002) and the complex social pressures of balancing the costs of pests evading detection versus the costs of consignments being rejected (See, 2012). Visual phytosanitary inspection at the border remains a critical component of the biosecurity system that serves as the last barrier where quarantine pests associated with imported commodities could be intercepted before entry into the importing country. As such, understanding the level of protection provided by consignment sampling and visual inspection is integral to any country's biosecurity system.

The cognitive processes involved in visual searching and detection of targets are complex and have been the subject of substantial research (Nakayama & Martini, 2011; Wolfe, 2018, Wolfe, 2020). Locating targets involves both top-down, task-driven and bottom-up, stimulus-driven elements, the relative importance of which have been the subject of extended investigation (Henderson, Malcolm, & Schandl, 2009; Parkhurst, Law, & Niebur, 2002; Underwood, Templeman, Lamming, & Foulsham, 2008). Identification of targets is a process that occurs in parallel with finding them and the factors which influence these two are difficult to disentangle (Godwin, Walenchok, Houpt, Hout, & Goldinger, 2015; Kotowicz, Rutishauser, & Koch, 2010). The ubiquity and versatility of our visual systems have made them of great importance for locating and identifying problems in various systems, but these visual inspection methods are subject to various sources of error (See, 2012). The reliability of visual inspection processes has been reported in fields such as finding flaws in aircraft parts (Aust, Pons, & Mitrovic, 2022) and nuclear weapons (See, 2015), aircraft safety (Drury & Prabhu, 1996) and flood defenses (Klerk, Kanning, Kok, Bronsveld, & Wolfert, 2021). Despite the importance of visual examination methods to phytosanitary operations, few studies have attempted to quantify the detection rates of pests or pathogens on imported commodities using visual methods. Neither has much research gone into ascertaining the relationship between the prevalence rate and detection probability (Perrone et al., 2013). Previous work on the detection efficacy of phytosanitary operations (e.g., Liebhold, Brockerhoff, Garrett, Parke, & Britton, 2012; Work, McCullough, Cavey, & Komsa, 2005) has considered the overall rate of detections by inspections when compared against the establishment of organisms of interest, as opposed to the reliability of the inspections themselves.

Several factors are known to affect the accuracy of visual inspection processes. These include the appearance of the objects, the environment in which the inspections are carried out, and the inspectors’ health and psychological state (See, 2012). Of particular importance is the attribute of saliency, being the degree to which the target (being pest organisms in the biosecurity context) differs from the background. The theory of signal detection (Sumner & Sumner, 2020) provides a framework for describing responses to perceptual stimuli.

Consequently, there is a need for research to assess the accuracy of visual inspections in detecting non-biosecurity–compliant goods and to understand the effects of prevalence rate, pest size, and color contrast with the host in the accuracy of such inspections. To this end, we designed experiments to test the effects of these variables of target saliency on the efficacy of visual inspections of fruit in a biosecurity context.

## Methods

### Participants

Eleven staff (three male, eight female) from The New Zealand Institute for Plant and Food Research Limited (PFR) of similar experience participated in these experiments. Vision and color-blindness tests of the inspectors were performed on the same day before the beginning of experimental work. The same inspectors were used across the whole experiment, carried out from September to December 2020. This research complied with the American Psychological Association Code of Ethics and the New Zealand National Ethics Advisory Committee Kāhui Matatika o te Motu National Ethical Standards for involving human participants. Each participant was fully informed about the purposes and aims of the research and gave their consent to be involved in the study.

### Stimuli

#### Model fruit

**Figure 1.**
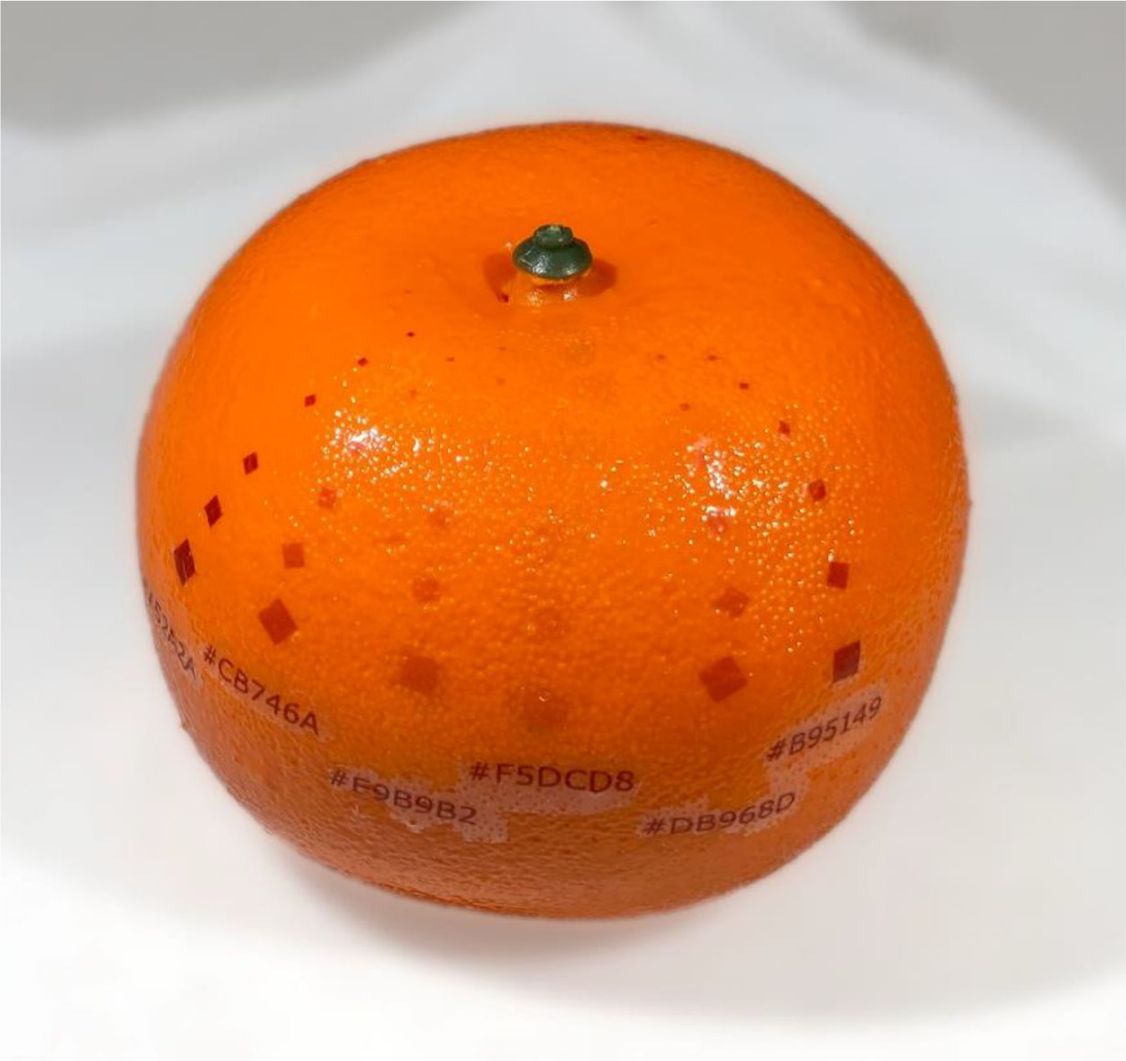
Example of the appearance of the decals used as targets and the model fruit on which they were affixed. From left to right, the six shades of brown used for the decals are #A52A2A (the darkest shade), #CB746A (darker mid-tone), #E9B9B2 (pale), #F5DCD8 (the palest shade), #DB968D (paler mid-tone), and #B95149 (dark), as labeled below each row of decals. The six sizes decrease from a square of 3 mm edge length on the outside, through edge lengths of 2 mm, 1.5 mm, 1 mm, and 0.7 mm to a 0.5 mm edge length square towards the center of the fruit. Note that only a single decal was affixed to the models examined by inspectors during the experiments.

To simulate the experience of phytosanitary inspections, 8000 model mandarin fruits made from expanded polystyrene (dimension: 6.5 × 7 × 4.5 cm) (model number APPLE 027; Yiwu United Crafts Co., Jinhua, China) were sourced for use as the items to be examined (Figure 1). By using artificial models, as opposed to natural fruits, we achieved relative homogeneity in the shape and size of the examined items.

#### Decals

To simulate the presence of pests on fruit, static marks were placed on models as the targets for inspectors to find. Living insects were not used as they could be dislodged or displaced during handling, skewing the results (Perrone et al., 2013). Preliminary tests used permanent marker pens with different colors and stencils to place circle-shaped marks of various sizes on the models. However, marks applied this way were inconsistent in their shape and color and thus deemed unsuitable. Instead, marks were applied using waterslide decals, which enabled greater consistency in the size and coloration.

Squares were created using Inkscape 0.92.4 (http://inkscape.org) with edge lengths of 3 mm, 2 mm, 1.5 mm, 1 mm, 0.7 mm and 0.5 mm and colored with a perceptually uniform color ramp of six shades of brown (RGB hexadecimal codes of #A52A2A [a dark brown], #B95149, #CB746A, #DB968D, #E9B9B2, and #F5DCD8 [a very pale brown]), chosen with the colorRampPalette() R function (Figure 1). The designs were printed onto waterslide decal paper (Ascent Print, Auckland) using a laser printer (Canon imageRUNNER ADVANCE C2030; Canon Inc., Tokyo, Japan) and then sealed with two coats of Liquid Decal Film (Microscale Industries Inc., Fountain Valley, CA, USA). Decals were cut out, soaked in water to remove the decal and then mounted onto the models using Micro Set (Microscale Industries Inc.). Excess mountant was removed using a cotton bud, and Micro Sol (Microscale Industries Inc.) was applied so that the decal conformed to the model's surface texture.

#### Color contrast measurement

The contrast between the marks and the background color of the model mandarins was measured using color photographs of marked models. Photographs were taken of marked models using a D5100 single-lens reflex camera (Nikon, Tokyo, Japan) mounted on a Kaiser RS2 copy stand (Kaiser Fototechnik GmbH & Co.KG, Buchen, Germany). Models were placed on a black velvet background and oriented such that the mark was facing the camera. Illumination was provided by two D-Lite RX One flash units (Elinchrom SA, Renens, Switzerland) fitted with Portalite softbox diffusers. The camera was operated using ControlMyNikon v. 4.3 (Nikon, Tokyo, Japan), with images saved in Joint Photographic Experts Group format, with dimensions of 3264 × 4928 pixels, and with three color channels (red, green, and blue, RGB). Images were captured in a single session.

The color of marks was measured from each image using FIJI (Schindelin et al., 2012) (http://fiji.sc). The mean values for each RGB channel were recorded from a manually defined region of interest (ROI) encompassing the mark. In addition, ten ROIs (100 × 100 pixels) were randomly placed throughout the area of the photograph covered by the model. RGB values were converted into the L* a* b* color space (Carter et al., 2018) using the ‘colorspace’ library in R (Zeileis et al., 2019). The luminance (L*) parameter of each ROI was used to calculate the Weber contrast ($C=\frac{L_{b}-\;L_{m}\;}{L_{b}}$) between the mark (*L_m_*) and the background (*L_b_*).

#### Prevalence rates

We tested if the prevalence rate per sample unit inspected for each mark–size combination played a role in visual inspection efficacy. The rates investigated were a low (1%–3%) and medium (8%–10%) prevalence rate. This equated to one to three and eight to 10 models marked in every sample of 100 units, respectively, with a total of 390 models marked out of the 7700 models used in the experiments. The precise number of models marked within each unit was randomly allocated using a random number generator. Five sample units with 0% prevalence rates were provided to inspectors for examination as operational controls, but these were not included in the final analysis of the data.

### Procedure

#### Inspection method

Models were assigned to sample units that consisted of bags containing 100 models. Each model was labeled with its bag and within-bag number. Randomly selected models were marked with the appropriate decal. Decal application and model numbering was completed by staff not involved in inspecting the models to avoid familiarizing the inspectors with the marks and their characteristics. Decals were applied haphazardly, with no intentional bias toward placing them in a particular location on the model.

Inspectors were trained before starting the trial by being instructed to examine the models thoroughly and showing them exemplar models and a printed sheet of the decals. These examples were available for inspectors throughout the trial. Inspectors examined each unit individually from the calyx end to the stalk end and over the back to the calyx (latitudinal rotation), then inspected the circumferences (longitudinal rotation). The environment in which inspections were performed conformed with phytosanitary inspection regulations, which state that during inspections, there should be a minimum of 1-m clear floor space separating each item, including (but not limited to) benches, boxes of plants or produce, desks, pallets of plants or plant material, quarantine bins, and tables (MPI, 2015; MPI, 2016). At the beginning of each trial, inspectors were given all the sample units they would inspect during that period. They were asked to record the time and the order in which they inspected the sample units. When examining a sample, two empty bags were provided to the inspector; one in which to place the models suspected to have decals and one to hold those deemed to be unmarked. Inspectors were asked to indicate the location of the decal by drawing an arrow using a whiteboard marker next to the identified decal. This allowed post-inspection assessments to evaluate whether the inspector had correctly located the decal or had incorrectly located a color defect on the model. After the sample unit had been examined, the inspectors returned all the unmarked models into the original bag and placed the bag containing marked models on the top.

The inspection protocols and standard requirements for operational conditions (e.g., light intensity) were developed through consultation with a government regulatory agency (New Zealand Ministry for Primary Industries, Plant Pest & Emerging Risk Assessment team) and an independent biosecurity consultant (SPS Biosecurity & Environmental Services Ltd). This ensured that the process followed was comparable to operational assessments of imported fresh produce.

#### Inspection trials

Inspectors examined sample units, each consisting of a bag filled with 100 models, a small proportion of which were marked with decals of a particular combination of size and color. During each inspection round, all 77 sample units were distributed among the 11 inspectors, with any one inspector examining seven sample units over the course of a three-hour session (13:00 to 16:00 hours local time). Each inspection round was carried out over two consecutive days, and inspectors were randomly assigned to the two days of the inspection round; about six inspectors were present in the laboratory during any particular session. Bright lighting over the inspection area was monitored using the mobile application Light Meter (Lux Light Meter Pro, version 2.1.1) to be at a minimum of 1000 lux on silver-colored tables, approved for color contrast.

A total of five inspection rounds were carried out over the duration of the experiment. At the end of the experiment each sample unit had been examined five times. Each inspector had examined a total of 35 sample units through the experiment, with no repeat assessments. The allocation of sample units to inspectors within and across inspection rounds was designed to account for carry-over effects, such as learning to recognize the artificial marks by the same inspectors. On completing an inspection round, the models selected by the inspectors were assessed to evaluate whether they were indeed marked and if the marks had been correctly identified. The arrows placed by inspectors to indicate the identified marks were removed and the decals checked to ensure they were suitable for the next inspection round. Any damaged or faded decals were replaced. Otherwise, sample units were unchanged between inspection rounds.

### Data analyses

The results of the examination of the marked models were classified into the following categories:

• Hit (*H*) = number of marked models selected by the inspectors with the decal correctly indicated
• Right fruit wrong reason (*W*) = number of marked models that were selected by the inspectors, but the decal was not recognized by the inspector
• Miss (*M*) = number of marked models incorrectly passed over by the inspectors
• False alarm (*F*) = number of unmarked models incorrectly selected by the inspectors
• Correct rejection (*C*) = number of unmarked models correctly passed over by the inspectors

Performance metrics were calculated on each sample unit from the number of model fruit in each of the above categories. In particular, the following metrics were calculated (Olson & Delen, 2008):

• Recall (Proportion of marked fruit that was correctly identified) = $\frac{H}{H\;+\;W\;+\;M}$
• Precision (Proportion of chosen fruit that were actually marked) = $\frac{H}{H\;+\;F}$
• Lucky guess rate = $\frac{W}{H\;+\;W\;+\;M}$
• False positive rate = $\frac{F}{F\;+\;C}$
• False negative rate = $\frac{M}{H\;+\;W\;+\;M}$

The signal detection metrics *d′* and *β* were calculated using the psycho R package (Makowski, 2018).

Binomial generalized linear mixed models (GLMM) were used to analyze how the treatment factors (i.e., mark size, mark contrast) influenced the performance measures described above. These mixed models included trial, inspector, and trial × inspector as random effects. A binomial generalized linear model was used to analyze the probability of selecting at least one marked model in a sample unit, using the prevalence rate as the predictor variable. All analyses were conducted using R (R Core Team, 2023), with the package glmmTMB (Magnusson et al., 2019) used to fit the GLMMs. Data have been made publicly available at FigShare and can be accessed at https://doi.org/10.6084/m9.figshare.26300920. This study was not preregistered.

## Results

### Target saliency

**Figure 2.**
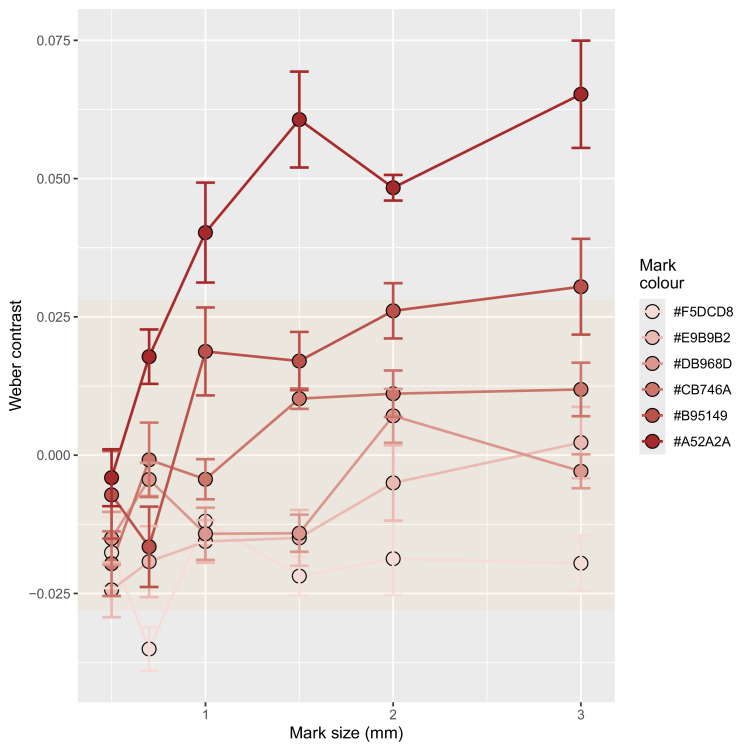
Mean and standard errors of the Weber contrast value for each mark size and color combination. Orange background shading indicates the variability (one standard deviation from 0) in the contrast values measured from randomly selected background regions.

The Weber contrast between the marks and the background color of the models varied from −0.035 (appearing paler than the background) to 0.065 (appearing darker than the background). The randomly selected samples of the background color had a mean standard deviation of 0.028. There was a significant interaction between mark size and mark color (*F* = 6.096, *df* = 25, *p* < 0.0001). The contrast of the marks against the background decreased with mark size, even when the marks were nominally the same color. Larger marks (>1 mm edge length) had a greater contrast, with the luminance of the darker colors being strongly different from the background (Figure 2).

### Inspector accuracy and performance

**Figure 3.**
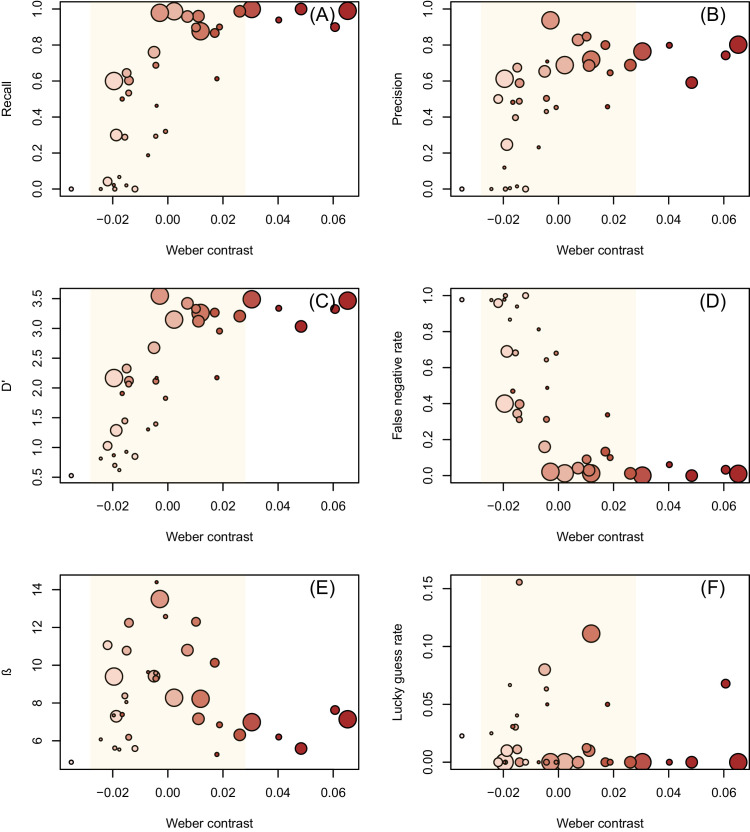
Inspection accuracy and performance measures and their relationship with the contrast of the target decals on the model fruit examined by inspectors. (**A**) Recall. (**B**) Precision. (**C**) D′ metric of signal strength. (**D**) False-negative rate. (**E**) The β measure of bias. (**F**) Lucky guesses. The size and color of points show the size and color of the target marks. Orange background shading indicates the variability (one standard deviation from 0) in the contrast values measured from randomly selected background regions. Note that the scale of the y-axis is the same in **A**, **B**, and **D** but differs in **C**, **E**, and **F**.

Recall varied from 0% to 100%, with the probability of mark detection increasing both when marks got larger (*p* < 0.001) and when they became darker relative to the background (*p* < 0.001) (Figure 3A). These relationships were additive, and the interaction between these two parameters was not significant (*p* = 0.77). Decals with an edge length larger than 2 mm had a mean recall that exceeded 90% across all colors except for the two palest shades, which were found with a mean recall between 20% and 70%. In contrast, the mean recall for models with decals smaller than 1 mm was 20%, although this was strongly skewed by relatively high detection of the darker colors of these sizes. When these were excluded, models marked with the four paler shades of decals less than 1 mm edge length had a mean recall of 9%.

Smaller marks needed to be darker relative to the background to be detected. The GLMM fitted to the recall data predicted that the Weber contrast at which recall would drop below 50% would increase as mark size decreased (*p* < 0.0001). Marks of 3-mm edge length were estimated to require a contrast of −0.02 before recall dropped below 50%, whereas this threshold contrast value increased to −0.004 for 2-mm marks, 0.004 for 1.5-mm marks, 0.12 for 1-mm marks, 0.017 for 0.7-mm marks, and 0.02 for 0.5-mm marks. These threshold contrasts all fell within 1 standard deviation of the background contrast variability.

The overall mean precision for these experiments was 40%, which varied between 0% and 80% across the size and color combinations. Sample units with larger, darker marks again received higher mean precision values (*p* > 0.001 for both size and contrast) (Figure 3B). However, in this case, an antagonistic interaction between the size and color of the decal was significant (*p* = 0.015), resulting in the largest, darkest marks having a lower precision than expected.

A similar result was obtained for the *d′* metric, which ranged between −0.49 and 4.48, with a mean of 2.19. Likewise, it had significant positive relationships with both size and color (*p* > 0.001 for both size and contrast) (Figure 3C) and had a significant (*p* > 0.001) but antagonistic interaction between them.

False-negative rates showed a pattern that was essentially the inverse of recall; sample units with a Weber contrast less than 0 had a much greater rate of false negatives (Figure 3D). However, even for sample units with marks in the three darkest colors and three largest sizes, there was a mean false-negative rate of 3.3%.

The *β* metric for bias ranged between 0.28 and 51.64 at the sample unit scale, with an average of 8.93. High values (>1 on an open-ended scale) indicate that inspectors were more likely to miss targets or say models were not marked. In contrast, low values (between 0 and 1) indicate that inspectors were more likely to claim that models did possess a mark. When grouped into combinations of size and color, the mean *β* for each combination ranged between 4.87 and 14.39. There was no significant relationship between *β* and size or color (size, *p* = 0.26; color, *p* = 0.91) (Figure 3E).

Lucky guesses occurred when inspectors returned models that had been marked, but the evidence they indicated for this decision was not the decal which had been applied to the model. These lucky guesses were more frequent for smaller, paler marks (Figure 3F) but were never frequent. On average, the lucky guess rate was only 2%, and the maximum rate of lucky guesses for a particular size/color combination was 15% (Figure 3F).

Across all response parameters, inclusion of a random effect to take into account the differing performance of the individual inspectors improved the GLMMs significantly (*p* = 0.0015, with a variance of 0.56 in the GLMM for recall), whereas the addition of random effects taking into account inspection round and the inspection round × inspector interaction did not improve the GLMMs.

### Prevalence rate

**Figure 4.**
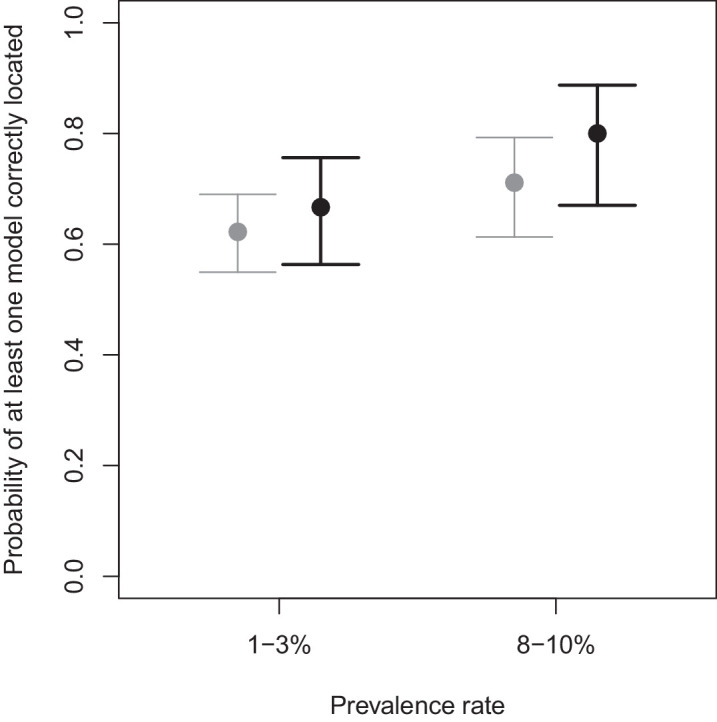
Mean and 95% confidence intervals of the probability of at least one marked model being correctly identified within a sample unit across the low (one to three marked models in a sample unit of 100 models) and high (eight to 10 marked models in a sample unit) prevalence rates investigated. Gray points show the overall mean across all combinations of size and color. Black points show the intermediate combinations (i.e. the three darkest colors of the three smallest mark sizes and the three palest colors of the three largest mark sizes).

We examined the impact of the prevalence rate on the probability of the inspectors correctly locating at least one marked model within a sample unit (bag of 100 models) to simulate the scenario of a biosecurity inspector finding a single insect in a shipment; a circumstance that would be sufficient to initiate mitigation measures. Overall, the prevalence rates examined in these experiments had no significant effect (*p* = 0.07) on whether a marked model was found, with a mean probability of 67%. However, for sample units containing intermediate marks (i.e., with neither the largest, darkest marks nor the smallest, palest marks), the higher prevalence rate resulted in an increase (80%) in the probability of at least one marked model in the sample unit being recognized, compared with a 67% chance of at least one model being recognized in a sample unit with a lower prevalence rate (Figure 4). There was no significant difference in the probability of finding at least one marked model between prevalence rates for those sample units that contained small, pale marks (mean probability = 22%), or those that had large, dark marks (mean probability = 98%). The sample units with 0% mark prevalence had an overall mean false-positive rate of 56% across all inspection rounds of the experiment, where at least one unmarked model in a sample unit was incorrectly considered by inspectors as possessing a mark.

### Timing effects

Two categories of misclassification decreased through the experiment, indicating that the inspectors improved as they because more familiar with the task. The incidence of lucky guesses declined from 4.62% in Inspection round 1 to 1.54% in Inspection round 5 (Table 1), whereas the false-positive rate declined from 6.10% in Inspection round 1 to 3.05% in Inspection round 5 (Table 1). In contrast, the false negative rate did not show a decline over time, with a mean of 42.1% over all five inspection rounds (Table 1), and the value of *β* increased from 7.2 in Inspection round 1 to 11.0 in Inspection round 4 (Table 1).

**Table 1.** Misclassification rates per inspection round. *Notes*: False-positive rate = percentage of unmarked models that were selected by inspectors; False-negative rate = percentage of marked models not selected by inspectors; Lucky guess rate = percentage of marked models that the inspectors selected, but where the feature indicated was not the mark applied to the model; β measure of bias = Values greater than 1 indicate that inspectors have a greater propensity to miss marked models.

Misclassifications	Round 1	Round 2	Round 3	Round 4	Round 5
False-positive rate	6.10	4.30	3.98	2.26	3.05
False-negative rate	37.9	45.9	42.6	44.6	39.5
Lucky guess rate	4.62	2.05	1.28	2.31	1.54
β measure of bias	7.2	8.1	9.3	11.0	9.1

False positives for sample units in the 0% prevalence sample units were very high in the first inspection round, with all five units having at least one model incorrectly considered as being marked (100% false-positive rate). This dropped to 3/5 (60%) in inspection rounds 2 and 3, 1/5 (20%) in inspection round 4, and 2/5 (40%) in inspection round 5. This decrease reflected the false-positive rate across all sample units (Table 1).

**Figure 5.**
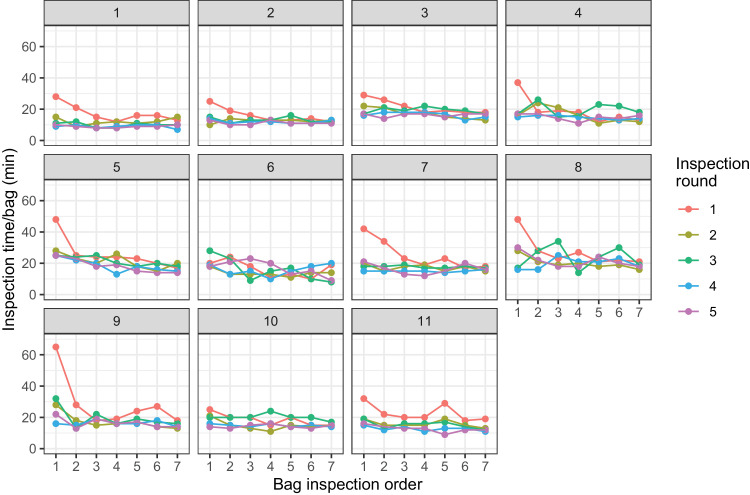
Time taken by inspectors to examine sample units of 100 models, plotted against the order in which they were processed during each inspection round. The five inspection rounds are plotted in different colors. Each facet represents an individual inspector.

On average, inspectors took 20 minutes to examine all 100 models in a sample unit (Figure 5). Inspectors took substantially longer to inspect the fruit in the first two sample units of Inspection round 1. However, by the end of that inspection round, inspection times had dropped to a level that the inspectors maintained in future inspection rounds. The time spent on each sample unit and the number of misidentifications reduced from the first to the seventh sample unit per inspector and within each inspection round.

Analysis of variance of the log-transformed inspection time, with explanatory factors of inspection round, inspection order, and the interaction between these two and a random effect for the inspector, indicated that the main effects and the interactions were all significant (*p* < 0.001). The interaction between the inspection round and inspection order is mainly driven by the time taken for the first and second sample units of the first inspection round.

## Discussion

Despite the importance of visual inspection processes for biosecurity purposes, relatively few studies have examined their efficacy. Many studies that have considered the problem focused on detecting pest organisms in the field (Arndt et al., 2022; Hammond, Hardie, Hauser, & Reid, 2016; Sheffield & Dugdale, 2020). The results of field surveillance experiments, which placed targets within a 12,500 m^2^ area, indicated that the overall probability of people finding targets within the environment was around 34%, with little difference between people considered experienced and novices (Mangano et al., 2011). However, there is a distinct lack of research into the ability of phytosanitary visual examination of fresh produce to detect unwanted organisms.

Our results demonstrate that large marks (>2-mm edge width) that strongly contrast with the background color are detected with high accuracy by inspectors. Smaller marks, or marks that contrasted poorly with the background, resulted in lower detection accuracies. In the case of the smallest, palest marks, the recall was very low (<10%). Our research also indicates that marks with an edge width of 1 mm or smaller and a Weber contrast lower (i.e., paler) than 0.012 from the background become increasingly difficult for inspectors to detect. These smaller, lower-contrast marks have comparable parameters to real-world examples of insects found upon harvested fruit. For example, the economically important pest scale insects *Hemiberlesia lataniae* and *H. rapax* form small (<1.5 mm diameter), circular waxy covers of the surface of many fruits, including kiwifruit, upon which they have a Weber contrast of 0.02 (Morales, 1988; SDJ Brown, unpublished data, 2023). Future pest risk assessments may do well to consider the contrast (and the contrast × size interaction) a pest may have against the goods transported in the risk pathway to evaluate the suitability of visual inspection of those goods for biosecurity purposes.

The selected mark size and color gradient resulted in expected variation in detectability by the naked eye when moving across scales from the small size and paler color towards darker and larger marks (Figure 4). The overall trend of detectability versus mark size and color shade suggests that the smaller sizes were mainly missed. However, when looking at the color shade change within the same mark size, inspectors could more easily detect the darker colors compared with the paler ones. A similar pattern was seen for color shades across the range of mark sizes (Figure 4). This corroborates other findings that there is an interaction between target size and salience on the accuracy of searches (Nuthmann, Clayden, & Fisher, 2021).

The prevalence rates tested here are likely substantially higher than the actual infestation rates of most imported commodities that require biosecurity inspections. Other research has shown that targets with low prevalence are more frequently missed during visual searches than targets with higher prevalence (Wolfe, Brunelli, Rubinstein, & Horowitz, 2013; Wolfe, Horowitz, & Kenner, 2005), although even rare targets can be located with high accuracy if the targets are clearly distinguishable (Biggs, Adamo, & Mitroff, 2014). Our results suggest that the detection probability of a model with a decal mark was unrelated to the prevalence rate per sample unit, instead indicating that mark size and color contrast to the model are more important parameters for their detection. However, the difference between the two prevalence rates we tested was much smaller than the rates tested in these previous studies, which may have prevented this effect from being shown clearly. As such, further research into the effect of prevalence rate into the likelihood of detection is warranted, especially where there are concerns that the threshold abundance for a pest to establish a new population might be lower than the threshold numbers for their detection by inspection (Memmott, Craze, Harman, Syrett, & Fowler, 2005; Simberloff, 2009).

The decreasing incidence of lucky guesses and false-positive rates through the experiment reflects the inspectors’ increased familiarity with the inspection method and the appearance of the decals used to mark the fruit. Similarly, the increasing value of *β* indicates the increased conservatism on the part of the inspectors. The inspectors were given feedback before the start of the inspections, had reference material on hand for examination during inspections, and were asked only to select the models they were confident had a decal. These factors will have led to the inspectors quickly learning to recognize what the decals looked like when they saw them. However, the false-negative rate remained constant across all inspection rounds, suggesting that these arose from the inspectors not perceiving the decals.

In addition to the prevalence rate and target salience tested here, several other factors will influence the efficacy of phytosanitary visual inspections. Inspector training and experience are well-known factors that can be readily remediated. In contrast, the effects of fatigue and the vigilance decrement (Al-Shargie et al., 2019) on inspection accuracy are poorly known in the biosecurity context and would benefit from further research. These experiments tested a single, simplified, immobile shape in various color shades, whereas in reality biosecurity inspectors have to be alert to the possibility of differently-shaped and potentially mobile targets. Experiments in other domains indicate that searching for multiple targets can decrease overall accuracy (Biggs, 2017; Cheng & Rich, 2018), and it would be interesting to examine the extent to which this phenomenon operates in the biosecurity context. In addition, bias is known to be affected by the relative severity of misses or false alarms (Lynn & Barrett, 2014), which can be significant in biosecurity operations. Further investigation of how inspectors modify their behavior under different understandings of the possible outcomes would be useful.

During operational biosecurity inspections in New Zealand, a sample of the consignment being imported is selected and examined for pests or other phytosanitary conditions (MPI, 2015). In many cases, detection of a single actionable pest insect would result in the importer being given the options of either undergoing additional phytosanitary treatments (e.g., fumigation) to kill any insects that are present, or to reship the consignment to a different market, or to have the consignment destroyed (MPI, 2015). Depending on the insects involved, a find on a consignment may instigate further upstream measures to make the pathway more secure by specifying actions (e.g., additional pest management practices or phytosanitary treatments) that importers or growers need to follow to ensure that unwanted organisms are not introduced through these trade pathways (Jamieson et al., 2022).

Although these experiments were primarily designed to answer applied questions of relevance to biosecurity operations, these results also inform theories about the relative importance of top-down or bottom-up influences on visual searches. Bottom-up theories predict that more strongly salient features capture attention of searchers (Henderson et al., 2009), whereas top-down theories argue that this attention capture is readily overridden by external guidance which directs viewers to ignore more salient distractors and focus on finding relevant features (Underwood et al., 2008). Our research demonstrates that a minimum salience is required for searchers to perceive the target, regardless of the top-down influences encouraging them to search for it.

From this research, we expect phytosanitary inspections will detect with higher probabilities larger pest species which contrast substantially with the commodities being examined. However, smaller pest species, especially those similar in coloration to the base color of the produce being examined, will be challenging to find in phytosanitary detections and have a higher chance of being missed. For these reasons, these properties should be considered during assessments of pest risk profiles and import pathways. Our results also demonstrate that any assumptions that biosecurity visual inspections are 100% accurate are misleading. Visual biosecurity inspections will remain an important measure to protect countries from invasive species. However, a greater appreciation of their limitations and challenges will help ensure they are utilized to their greatest potential and efficiency.

## Conclusions

Detection of marks on model fruit in experiments designed to test the accuracy of visual inspections by manual inspectors in a biosecurity context was heavily influenced by the size and the color of marks. Although large, dark marks were found with high recall, small, low-contrast marks were infrequently found. In general, inspectors get better at knowing what targets look like over time, but if they cannot perceive the target, they cannot act upon it.
